# Variation for Photoperiod and Temperature Sensitivity in the Global Mini Core Collection of Sorghum

**DOI:** 10.3389/fpls.2021.571243

**Published:** 2021-06-29

**Authors:** Hari D. Upadhyaya, M. Vetriventhan, Vania C. R. Azevedo

**Affiliations:** ^1^Genebank, International Crops Research Institute for the Semi-Arid Tropics (ICRISAT), Patancheru, India; ^2^Plant Genome Mapping Laboratory, University of Georgia, Athens, GA, United States

**Keywords:** sorghum, photosensitivity, germplasm, photoperiod insensitive, cumulative growing degree days

## Abstract

Information on photoperiod and temperature sensitivity of sorghum germplasm is important to identify appropriate sources for developing cultivars with a broad adaptation. The sorghum mini core collection consisting of 242 accessions along with three control cultivars were evaluated for days to 50% flowering (DFL) and plant height in two long-day rainy and two short-day post-rainy seasons, and for grain yield and 100-seed weight in the two post-rainy seasons. Differences in DFL and cumulative growing degree days (CGDD) in the rainy and post-rainy seasons were used to classify the accessions for photoperiod and temperature sensitivity. Results revealed 18 mini core landraces as photoperiod and temperature insensitive (PTINS), 205 as photoperiod sensitive and temperature insensitive (PSTINS), and 19 as photoperiod and temperature-sensitive (PTS) sources. The 19 PTS sources and 80 PSTINS sources took less DFL in the long-day rainy seasons than in the short-day post-rainy season indicating their adaptation to the rainy season and a possible different mechanism than that trigger flowering in the short-day sorghums. In all three groups, several accessions with desirable combinations of agronomic traits were identified for use in the breeding programs to develop climate-resilient cultivars and for genomic studies to identify genes responsible for the photoperiod and temperature responses.

## Introduction

Sorghum [*Sorghum bicolor* (L.) Moench] is an important multipurpose crop grown for food, feed, fodder and fuel purposes. It is a C_4_ crop with high photosynthetic efficiency, cultivated in both temperate and tropical regions between 40°N to 40°S of the equator (Doggett, 1988). Sorghum ranks fifth among the cereal crops after maize (*Zea mays* L.), rice (*Oryza sativa* L.), wheat (*Triticum aestivum* L.), and barley (*Hordeum vulgare* L.). During 2018, sorghum was grown on an estimated area of 42.14 Mha with a production of 59.34 Mt in over 100 countries, with a productivity of 1408 kg ha^–1^. Globally, sorghum is largely cultivated during the rainy season in diverse conditions which is often affected by moisture stress and also several diseases and insect pests affect crop productivity (Assefa et al., 2010; Upadhyaya et al., 2019). In India sorghum is grown in two distinct seasons: kharif (rainy season, June–October) and rabi (post-rainy season, October–February). Kharif season is characterized by long-days with high temperature and rabi season by the short-days with low temperature. It is therefore important to develop high-yielding cultivars resistant to various biotic and abiotic stresses and adapted to diverse climatic conditions.

Variation in the germplasm collection is key to crop improvement. Vast germplasm collections are conserved in the national and international Genebanks. Globally over 23,6000 accessions of sorghum have been conserved in the genebanks having a rich diversity for various traits of economic importance (Upadhyaya et al., 2016b). The ICRISAT Genebank has the largest collection of over 41,000 sorghum accessions from 93 countries. Large size of germplasm collection and non-availability of reliable data on traits of economic importance restricted breeders from using germplasm collections and caused them to use their working collections (Upadhyaya et al., 2014d). This results in low use of germplasm in crop improvement and consequently a narrow genetic base of crop cultivars (Dalrymple, 1986; Vellve, 1992; Upadhyaya et al., 2014b). Genetic studies indicated that the sorghum wild and weedy relatives and landraces are more diverse than the improved hybrids (Muraya et al., 2011; Mace et al., 2013). For example, in the United States, the improved sweet sorghum lines were mostly derived from six landraces therefore the majority of the lines have similar genes/alleles for high Brix % (Murray et al., 2009). The genetic diversity assessment sorghum hybrids widely cultivated in the United States between 1980 and 2008 indicated that the hybrids released during the 2000s had the least number of new alleles as compared to the previous two decades (Smith et al., 2010). Similarly, a decline in genetic diversity was reported in Australian sorghum breeding programs as a result of a strong selection for resistance to the sorghum midge, stay-green (a drought resistance trait) and other agronomic traits (Jordan et al., 1998, 2003). In sorghum which is a short-day plant (SDP), several germplasm accessions do not flower or take much more time in the long-day rainy season and thus restrict the use of germplasm in crop breeding programs.

The germplasm diversity representative subsets such as core collection (10% of the entire collection) and mini core collection (10% of core collection or 1% of the entire collection) conserved in a genebank are the ideal genetic resources for identifying new sources of variation for traits of interest (Upadhyaya and Ortiz, 2001; Upadhyaya et al., 2013, 2014c). In this study, we evaluated 242 accessions of sorghum mini core collection (Upadhyaya et al., 2009) for photoperiod and temperature sensitivity in the two rainy and two post-rainy seasons at ICRISAT, Patancheru, India, to identify insensitive accessions that can be used in crop improvement to develop high-yielding photoperiod and temperature insensitive cultivars with a broad genetic base and greater adaptation.

## Materials and Methods

### Description of Materials

The 242 accessions in the sorghum mini core collection (Upadhyaya et al., 2009) and three controls (IS 33844, IS 18758 and IS 2205) were utilized in this study. The mini core accessions originating (collection site) from 57 countries, and it included all the five basic races (caudatum 16.1%, durra 12.4%, guinea 12%, kafir 8.7%, and bicolor 8.3%) and 10 intermediate races (caudatum-bicolor 12.4%; guinea-caudatum 11.2%; durra-caudatum 7.9%; durra-bicolor and kafir-caudatum each 2.9%; kafir-durra 1.7%; guinea-kafir 1.2%; and guinea-bicolor, guinea-durra, and kafir-bicolor each 0.8%) of sorghum (Upadhyaya et al., 2009). The two controls, IS 33844 and IS 18758 are the germplasm accessions and selection from these were released as cultivars. IS 33844 was released as Parbhani Moti in India, which is the most popular drought-tolerant cultivar grown under receding soil moisture conditions during the post-rainy seasons and possesses excellent grain quality attributes (Upadhyaya et al., 2017). The selection from IS 18758 was released in Burkina Faso as E-35-1 and in Burundi as Gambella 1107 (Upadhyaya et al., 2014a). IS 2205 is a durra-bicolor landrace originating from India is resistant to shoot fly [*Atherigona soccata* (Rondani)] and stem borer [*Chilo partellus* (*Swinhoe*)] (Sharma et al., 2003).

### Experimental Details

The experiments were conducted at the ICRISAT, Patancheru (17.51° N, 78.27° E, and 545 masl), India during the 2010 and 2011 rainy and 2010–2011 and 2011–2012 post-rainy seasons. The precision fields of the ICRISAT, Patancheru center having uniform fertility with a gentle slope of 0.5% were used. The experiments were conducted in an Alpha design in the rainy seasons with three replications. In the post-rainy seasons, accessions were planted in a split-plot design in three replications using drought stress and optimally irrigated treatments as the main plot and genotypes as the subplots in five maturity groups (Upadhyaya et al., 2017), and data from the optimally irrigated condition was used for this study. The experimental materials were planted in the last week of June in the rainy season and in the second week of October in the post-rainy season each year. Each accession was sown in a single row of 4 m long plot and the spacing between plots was 75 cm. Seeds were sown at a uniform depth of 2–3 cm using a tractor-mounted four-cone planter, and excess seedlings were thinned during 2 to 3rd weeks after sowing, by maintaining the plant-to-plant spacing of about 10 cm. The crop-specific agronomic practices such as weeding, thinning, irrigation and plant protection measures were followed. A basal dose of Ammonium phosphate was applied at the rate of 150 kg ha^–1^, and urea was applied as topdressing at the rate of 100 kg ha^–1^ during the 3 weeks after planting. A ridge and furrow system of cultivation was adopted, and each time, the experimental plots received about 7 cm of irrigation water. Observations on days to 50% flowering (DFL, the day when 50% or more of the plants had reached anthesis in a plot) were recorded in each of the two rainy and two post-rainy seasons. Plant height was recorded on five representative plants at maturity in the rainy and post-rainy seasons whereas data on grain yield and 100-seed weight was recorded only in the post-rainy season on five representative plants.

### Data Analysis

Data on daily minimum and maximum temperatures was obtained from the ICRISAT intranet^1^ and was used to calculate the growing degree days (GDD) in the two rainy and two post-rainy seasons separately.

The GDDs were computed using the following equation:

$$\begin{array}{l} \text{GDD}=[(\text{daily maximum temperature}+\text{daily minimum}\\ \text{ temperature})/2]-10^{\circ }\text{C},\text{ the base temperature}\\ \text{ for sorghum}]. \end{array}$$

The cumulative growing degree days (CGDD) were computed as follows:

CGDD = the sum of all daily GDD from the effective date of sowing to the date of 50% flowering.

For sorghum a base temperature of 10°C (Gerik et al., 2003) was used. Data on DFL, CGDD, and plant height in the two rainy and two post-rainy and grain yield and 100-seed weight in the two post-rainy seasons were analyzed separately using Genstat 17^2^ following residual (or restricted) maximum likelihood (REML) (Patterson and Thompson, 1971). Combined analyses of two rainy seasons and two post-rainy seasons were performed separately considering genotypes as random and seasons fixed. Variance components due to genotypes and genotype × environment (season) were estimated and tested for their significance against their respective standard errors. Wald’s statistic (Wald, 1943) for the season was calculated and tested to determine the significance of the two rainy or two post-rainy seasons. For DFL, CGDD, plant height, grain yield and 100-seed weight, BLUPs (balanced linear unbiased predictors) were obtained for combined analysis of two rainy and two post-rainy seasons, separately.

### Classification of Mini Core Accessions for Photoperiod and Temperature Sensitivity

The mini core accessions were categorized into photoperiod and temperature-sensitive/insensitive considering the differences in DFL and CGDD requirement during the long-day rainy season and short-day post-rainy seasons. When the difference between DFL in rainy and post-rainy seasons was equal to twice the standard error of the mean (±4 calendar days), the accessions were classified as photoperiod-insensitive while the remaining were considered as photoperiod-sensitive. Similarly, in the case of temperature sensitivity, the accessions were classified as temperature-sensitive when the difference in CGDD requirements in the rainy and post-rainy seasons was equivalent to ±4 calendar days (−57 to +57°d) while the remaining accessions on both sides (+ and −) were categorized as temperature-insensitive. Finally, both photoperiod and temperatures sensitivities were considered together which resulted in three groups of accessions: photoperiod- and temperature-insensitive (PTINS), photoperiod-sensitive and temperature-insensitive (PSTINS), and photoperiod- and temperature-sensitive (PTS).

The phenotypic correlations among DFL, CGDD, and plant height in both the rainy and post-rainy seasons and grain yield and 100-seed weight in the post-rainy season were calculated using Genstat 17 (see Text Footnote 2) to determine the relationships between traits in the entire mini core collection, PTINS, PSTINS, and PTS groups of accessions.

## Results

### Variance Components, Mean and Range

The REML analysis of individual rainy and post-rainy season data, and combined of two rainy and two post-rainy seasons revealed significant genotypic variance (*σ*^2^_g_) for DFL, CGDD, plant height, grain yield and 100-seed weight (Supplementary Table 1). The significant interactions between genotypes and seasons (*σ*^2^ ge) was observed for all the traits in both rainy and post-rainy seasons, except in group 5 where the plant height, grain yield per plant and 100-seed weight were not significant (Supplementary Table 1). Wald’s statistics was significant for all the traits in the rainy and post-rainy seasons except in the G5 in the post-rainy season for 100-seed weight.

In the mini core collection, the DFL ranged from 45 to 152 days in the 2010 rainy season and 42–142 days in the 2011 rainy season (Table 1). On average, mini core accessions flowered 10 days later in 2010 than in the 2011 rainy season indicating differences in the two rainy seasons mainly due to varying GDD per calendar day during flowering, besides other factors including the genotype by environment interactions. Averaged over two rainy seasons the DFL ranged from 44 (IS 12706) to 148 days (IS 15744) (Table 1 and Supplementary Table 2). The differences in the DFL have also reflected the differences in the CGDD in the two rainy seasons. The CGDD to flower ranged from 758 to 2,403°d in the 2010 rainy season with a greater mean (1,415°d) than in the 2011 rainy season (range 731–2,275°d, mean 1,297°d). Averaged over both the rainy seasons the CGDD varied from 756 (IS 12706) to 2,356 (IS 15744) with a mean value of 1,369°d (Supplementary Table 2). In the post-rainy seasons also the DFL differed between 2010–2011 and 2011–2012 seasons. The DFL ranged from 47 to 109 in the 2010–2011 and 51 to 132 in the 2011–2012 post-rainy season. On average mini core accessions took 5-days more to flower in the 2011–2012 season (75 days) than in the 2010–2011 season (70 days). Averaged over both the post-rainy seasons, DFL ranged from 51 days (IS 32245) to 120 (IS 31446) with an average of 73 days (Supplementary Table 2). The CGDD required to flower in the 2010–2011 post-rainy season were less (793°d) with a lower range (577 to 1,270°d) than in the 2011–2012 post-rainy season (mean 966°d, range 693 to 1,698°d). This indicated that the two post-rainy seasons affected the expression of flowering in the mini core collection accessions. On average over the two post-rainy seasons, the mini core accessions took 880°d to flower with IS 32245 flowering earliest requiring only 658°d and IS 31446 being the latest requiring 1,485°d. Both in terms of DFL and the CGDD requirements highly significant genotype × season (year) interaction was observed (Supplementary Table 1). Plant height of mini core accessions also showed a great deal of differential expression in the two rainy and two post-rainy seasons. On average plant height was higher in the 2011 rainy season (326 cm) than in the 2010 rainy season (281 cm) with a greater range in the former (117–501 cm) than the latter (126–406 cm) (Table 1). Similarly, in the 2011–2012 post-rainy season mean (256 cm) and ranges (99–390 cm) for plant heights were more than in the 2010–2011 post-rainy season (214 and 105–347 cm) (Table 1).

**TABLE 1:** Mean and range of different traits in the 2010 and 2011 rainy and 2010–2011 and 2011–2012 post-rainy seasons and combined of two rainy and two post-rainy seasons at ICRISAT, Patancheru, India.

Trait	Rainy 2010		Rainy 2011		Combined	
	Mean	Range	Mean	Range	Mean	Range
Days to 50% flowering (days)	86.6	45–152	77.1	42–142	83	44–148
Cumulative growing degree days (°d)	1415	758–2403	1297	731–2275	1369	756–2356
Plant height (cm)	281	126–406	326	117–501	301	115–446

**Trait**	**Post-rainy 2010–11**		**Post-rainy 2011–12**		**Combined**	
	**Mean**	**Range**	**Mean**	**Range**	**Mean**	**Range**

Days to 50% flowering (days)	70	47–109	75	51–132	73	51–120
Cumulative growing degree days (°d)	793	577–1270	966	693–1698	880	658–1485
Plant height (cm)	214	105–347	256	99–390	235	106–369
Grain yield per plant (g)	21	2–41	26	3–74	24	2–54
100-seed weight (g)	2.50	0.50–5.00	2.40	0.50–5.70	2.43	0.48–5.33

### Photoperiod and Temperature-Sensitive and Insensitive Mini Core Accessions

The criteria of twice the standard error of the mean for differences in DFL and CGDD requirements between the long-day rainy seasons (combined of two rainy seasons) and the short-day post-rainy seasons (combined of two post-rainy), the mini core collection accessions were classified into three groups: PTINS (18 accessions), PSTINS (205 accessions) and PTS (19 accessions) (Supplementary Table 2). The three control cultivars were also grouped into PTINS (IS 2205 and IS 33844) and PSTINS (IS 18758). On average, the PTINS took 57–84 days in the rainy seasons and 56–87 days in the post-rainy seasons and the differences in DFL of the 18 individual PTINS accessions between the rainy and post-rainy seasons were within ±4 days (Table 2) indicating their adaptation to both rainy and post-rainy seasons. The CGDD requirements of the PTINS accessions were more in the rainy season (1,159°d) than in the post-rainy season (837°d). For 18 PTINS mini core accessions individually, the CGDD requirement ranged from 971 to 1,386°d in the rainy seasons and 711 to 1,041°d in the post-rainy season and differences between two seasons ranged from 218°d (IS 4060) to 417°d (IS 7131). The two PTINS controls, IS 2205 and IS 33844 required to flower 1,267 and 1,239°d in the rainy season and 960 and 924°d in the post-rainy season, respectively.

**TABLE 2:** Performance of photoperiod and temperature insensitive landraces identified in the sorghum mini core collection, evaluated during two rainy and two post-rainy seasons at ICRISAT, Patancheru, India.

IS No.	Origin	Race	Days to 50% flowering		Cumulative growing degree days (CGDD)		Plant height (cm)		Grain yield (g)	100-seed weight (g)
			Rainy	Post-rainy	Rainy	Post-rainy	Rainy	Post-rainy		
473	United States	GK	64	66	1074	799	269	211	14	1.48
2864	South Africa	C	61	58	1025	728	189	137	20	2.14
4060	India	DB	57	61	971	753	274	194	16	2.09
4581	India	D	72	75	1201	893	286	226	33	2.63
4631	India	D	74	72	1233	857	309	244	31	2.89
5094	India	D	74	76	1223	905	335	272	30	2.45
7131	Uganda	DC	84	81	1386	969	299	231	27	2.24
11619	Ethiopia	DB	83	87	1369	1041	301	331	20	1.87
12697	Australia	B	76	72	1268	869	335	260	10	1.43
12937	Ethiopia	K	69	69	1151	830	331	245	24	2.04
14290	Botswana	KD	67	69	1120	833	297	238	25	2.41
17941	India	C	60	57	1009	717	185	129	16	2.39
20743	United States	B	67	63	1122	770	326	231	17	1.92
21645	Malawi	GK	68	65	1141	791	302	219	11	1.70
24348	India	C	59	56	1006	711	201	153	17	2.19
29239	Swaziland	K	68	71	1140	856	214	193	26	2.99
29568	Lesotho	KC	63	67	1069	806	292	237	29	2.55
29714	Zimbabwe	KD	82	78	1350	941	263	221	26	1.77
Mean			69	69	1159	837	278	221	22	2.20
Range			57–84	56–87	971–1386	711–1041	185–335	129–331	10–33	1.43–2.99
**Controls**										
2205	India	DB	76	80	1267	960	334	251	26	2.10
18758	Ethiopia	GC	78	69	1288	833	283	166	29	2.40
33844	India	D	75	77	1239	924	325	262	41	3.60
*SE*(*d*)			2.45	1.48	39.88	14.18	16.00	11.16	4.83	0.19
LSD (*P ≤* 0.05)			4.80	2.89	78.17	27.79	31.36	21.87	9.46	0.37
CV (%)			4.18	1.95	4.11	1.53	7.51	4.63	21.45	8.10

The PTS landraces flowered early in the rainy seasons and on average took 52 days compared to 72 days in the post-rainy season and showed similar CGDD requirement (±57°d). The DFL requirement of the 19 individual PTS accessions ranged from 46 to 66 days in the rainy season and 60 to 94 days in the post-rainy season (Table 3). The CGDD requirement was between 785 and 1,110°d in the rainy season and 738 and 1,121°d in the post-rainy season.

**TABLE 3:** Performance of photoperiod and temperature sensitive landraces identified in the sorghum mini core collection evaluated during two rainy and two post-rainy seasons at ICRISAT, Patancheru, India.

IS No.	Origin	Race	Days to 50% flowering		Cumulative growing degree days		Plant height (cm)		Grain yield (g)	100-seed weight (g)
			Rainy	Post-rainy	Rainy	Post-rainy	Rainy	Post-rainy		
1212	China	KB	47	65	801	788	255	238	17	2.38
1219	China	GB	47	64	803	779	261	241	22	2.00
16151	Cameroon	CB	48	68	819	816	238	242	22	1.64
19676	Zimbabwe	K	66	88	1106	1048	189	156	20	2.50
20727	United States	B	46	65	785	791	221	219	14	1.37
22616	Myanmar	B	62	88	1052	1054	293	287	15	1.33
24503	South Africa	B	49	73	839	880	259	248	2	0.48
26701	South Africa	CB	61	87	1031	1039	245	224	20	1.36
28614	Yemen	DC	49	66	828	798	282	240	18	2.15
29314	Swaziland	DC	66	92	1109	1097	298	278	22	1.87
29689	Zimbabwe	K	66	94	1110	1121	281	271	25	2.27
30383	China	CB	48	64	819	776	266	247	17	2.06
30400	China	CB	49	66	830	803	286	271	22	2.05
30417	China	CB	49	64	833	782	288	259	25	2.33
30450	China	CB	47	64	803	776	231	202	18	2.43
30451	China	CB	49	65	839	786	255	223	28	2.11
30466	China	CB	46	60	785	738	248	240	18	2.01
30507	Korea	CB	49	64	828	776	272	235	21	2.04
30508	Korea	CB	49	66	828	801	214	219	19	1.92
**Control**										
2205	India	DB	76	80	1267	960	334	251	26	2.14
18758	Ethiopia	GC	78	69	1288	833	283	166	29	2.37
33844	India	D	75	77	1239	924	325	262	41	3.60
Mean			52	72	887	866	257	239	19	1.91
Range			46–66	60–94	785–1110	738–1121	189–298	156–287	2–28	0.48–2.50
*SE(d)*			2.45	1.48	39.88	14.18	16	11.16	4.83	0.19
LSD (P *≤* 0.05)			4.8	2.89	78.17	27.79	31.36	21.87	9.46	0.37
CV (%)			4.18	1.95	4.11	1.53	7.51	4.63	21.45	8.10

The 205 PSTINS mini core accessions on average took 14 days and 548°d more in the rainy season (87 and 1,433°d) for flowering than in the post-rainy season (73 and 885°d) (Table 4). However, within these 205 mini core accessions, there were vast differences in terms of DFL and CGDD requirements in the rainy season *vis-a-vis* their requirements in the post-rainy season. The DFL ranged from 44 to 148 days in the rainy and from 51 to 120 days in the post-rainy season and the CGDD requirement from 756 to 2,356 °d in the rainy and from 658 to 1,485°d in the post-rainy season. This indicated different adaptation patterns of PSTINS accessions in terms of DFL and CGDD requirement for flowering. This group was therefore further categorized into three sub-groups. The accessions that took fewer days and fewer CGDD for flowering in the rainy season than in the post-rainy season were grouped into subgroup 1 following (Upadhyaya et al., 2018). None of the mini core accessions fell into this subgroup. The accessions that took fewer days and more CGDD in the rainy season than in the post-rainy accessions were grouped into Subgroup 2 and the accessions, which took more days and more CGDD in the rainy season than in the post-rainy were classified into Sub-group 3. In the mini core collection, 80 accessions were categorized into Subgroup 2 and 125 and control IS 18758 into Subgroup 3. The accessions in the Subgroup 2 on average took 11 days less in the rainy season (60 days) than in the post-rainy season (71 days) whereas those in the Subgroup 3 took 30 days more in the rainy season (104 days) than in the post-rainy season (74 days). Individually the difference between DFL in the rainy season and the post-rainy season ranged from −22 to −5 days in subgroup 2 compared to 5–74 days in the Subgroup 3. Of the 125 accessions in the Subgroup 3, 59 accessions took on average 30–74 days more, 24 took 45–74 days more and 7 took 61–74 days more in the rainy season than in the post-rainy season (Supplementary Table 2). Contrastingly, in Subgroup 2, 20 accessions took 15–22 days less in the rainy season than in the post-rainy season, indicating the differential response of these two subgroups for flowering. These accessions could be useful sources for the sorghum improvement program for the two distinct conditions, Subgroup 2 for the long-day rainy season and subgroup 3 for the short-day post-rainy season.

**TABLE 4:** Performance of 15 best yielding landraces identified in the photoperiod sensitive and temperature insensitive subgroups 2 and 3 in the sorghum mini core collection evaluated in two rainy and two post-rainy seasons at ICRISAT, Patancheru, India.

IS No.	Origin	Race	Days to 50% flowering		Cumulative growing degree days		Plant height (cm)		Grain yield (g)	100-seed weight (g)
			Rainy	Post-rainy	Rainy	Post-rainy	Rainy	Post-rainy		
**Sub-group 2 (80 accessions)**										
4698	India	D	70	78	1175	935	282	230	54	3.36
22294	Botswana	K	67	77	1128	926	298	285	36	3.12
29241	Swaziland	KC	59	73	1007	874	266	273	36	2.35
29187	Swaziland	GC	65	79	1093	951	278	256	35	2.11
13971	South Africa	C	65	77	1089	924	282	264	35	2.23
4515	India	D	70	75	1162	891	311	244	33	3.15
29627	South Africa	DC	56	69	952	827	288	249	33	2.27
19450	Botswana	GK	57	66	971	798	237	198	32	2.47
29606	South Africa	K	62	77	1053	937	310	264	31	2.96
29358	Lesotho	K	62	68	1044	816	249	202	30	2.39
2389	South Africa	K	56	68	949	815	233	219	30	2.56
29565	Lesotho	GC	57	66	963	798	242	221	30	2.84
30460	China	C	54	63	921	769	255	206	29	2.42
29582	Lesotho	K	62	69	1049	828	187	150	29	2.44
2872	Egypt	CB	48	60	822	743	181	147	25	3.61
Mean of top 15 accessions			61	71	1025	855	260	227	33	2.68
Range of top 15 accessions			48–70	60–79	822–1175	743–951	181–311	147–285	25–54	2.11–3.61
Mean of Sub-group 2			60	71	1009	862	250	220	24	2.28
Range of Sub-group 2			44–83	54–93	756–1369	685–1108	115–354	106–311	10–54	1.32–3.61
**Subgroup 3 (125 accessions)**										
1004	India	D	93	84	1539	1007	355	286	49	3.84
23590	Ethiopia	GC	82	72	1358	866	282	220	43	3.04
28141	Yemen	DC	103	75	1688	911	332	249	42	5.18
23891	Yemen	D	99	75	1623	909	394	270	42	5.33
27034	Sudan	D	113	89	1844	1058	365	317	41	3.42
23586	Ethiopia	GC	81	73	1339	874	289	221	40	2.94
31706	Yemen	D	108	74	1782	893	388	308	39	4.69
23514	Ethiopia	C	75	69	1243	826	262	190	38	2.81
21083	Kenya	C	108	77	1776	922	342	297	37	2.58
6421	India	D	109	80	1790	969	371	262	37	2.63
20632	United States	C	127	82	2065	985	390	326	34	3.10
4613	India	D	80	75	1328	896	350	229	32	2.99
19153	Sudan	GC	77	70	1280	836	247	165	30	2.45
9108	Kenya	C	77	71	1281	844	327	237	27	2.66
23579	Ethiopia	GC	77	67	1280	806	323	220	37	2.58
Mean of top 15 accessions			94	75	1548	907	334	253	38	3.35
Range of top 15 accessions			75–127	67–89	1243–2065	806–1058	247–394	165–326	27–49	2.45–5.33
Mean of Sub-group 3			104	74	1704	899	343	247	24	2.63
Range of Sub-group 3			61–148	51–120	1026–2356	658–1485	192–446	120–369	7–49	1.00–5.33
Mean of entire PSTINS			87	73	1433	885	307	236	24	2.99
Range of entire PSTINS			44–148	51–120	756–2356	658–1485	129–446	106–369	7–54	1.00–5.33
Control										
2205	India	DB	76	80	1267	960	334	251	26	2.14
18758	Ethiopia	GC	78	69	1288	833	283	166	29	2.37
33844	India	D	75	77	1239	924	325	262	41	3.60
*SE(d)*			2.45	1.48	39.88	14.18	16.00	11.16	4.83	0.19
LSD (*P ≤* 0.05)			4.80	2.89	78.17	27.79	31.36	21.87	9.46	0.37
CV (%)			4.18	1.95	4.11	1.53	7.51	4.63	21.45	8.10

The CGDD requirements of the Subgroup 2 was on average, 1,009°d in the rainy and 862°d in the post-rainy with a narrow range in both the seasons (756–1,369°d in rainy; 685–1,108°d in post-rainy). The CGDD requirements for the Subgroup 3 landraces was on average 1,704°d in the rainy and 899°d in the post-rainy season with a wide range in the rainy (1,026–2,356°d) and post-rainy (658–1,485°d). Individually the differences in the CGDD requirements in the rainy and post-rainy season of the 125 accessions in the Subgroup 3 were much greater (341–1,475°d) than in the 80 accessions of Subgroup 2 (58–278°d). This clearly established that the PSTINS accessions of the mini core in group 2 and 3 were different in their adaptation in terms of DFL and CGDD requirements.

### Agronomic Performance of Mini Core Accessions

The mini core accessions and three control cultivars, two PTINS (IS 205, IS 33844) and one PSTINS (IS 18758), were evaluated for grain yield per plant and 100-seed weight. Vast differences in the grain yield per plant were observed between accessions of the mini core collections in both the post-rainy seasons.

### Performance of PTINS Mini Core Sources

The detailed information on the performance of 18 PTINS sources and three control cultivars is given in Table 2. The PITNS originated from 11 countries, 6 from India, and 2 each from Ethiopia and the United States and 1 each from eight other countries (Table 2). Over both seasons, three PTINS produced a grain yield of 30–33 g per plant, however, none of them was superior to the IS 33844 (Table 2). For 100-seed weight also, none of the PTINS (1.43–2.99 g) was superior to IS 33844 (3.6 g) (Table 2). Considering DFL in the rainy and post-rainy seasons and yield per plant of IS 29568 appears promising.

### Performance of PSTINS Mini Core Sources

The 205 PSTINS accessions originated from 54 countries, six countries contributing 10 or more accessions were Cameroon (12), Ethiopia (10), India (24), Yemen (15), the United States (13) and South Africa (22). Nine accessions in the combined over two post-rainy seasons (39–54 g) performed very well and yielded significantly greater than the PSTINS control IS 18758 (29 g) (Table 4). For 100 seed weight, 68 lines had greater 100-seed weight than the IS 18758 (2.4 g).

Subdivision of PSTINS into Subgroups revealed adaptation of Subgroup 2 to the rainy season and Subgroup 3 to the post-rainy season in terms of the DFL and CGDD requirements. This was further confirmed by the performance of these two subgroups in terms of grain yield and 100-seed weight (Table 4). Though the overall performance of both the subgroups in terms of mean grain yield per plant was similar (24 g), the number of accessions that produced significantly greater yield than the control IS 18758 were higher (17 in 2010–2011, 13 in 2011–2012, 8 overall) in the Subgroup 3 compared to subgroup 2 (7 in 2010–2011, 3 in 2011–2012 and 1 overall). In terms of percentage also, since the number of accessions in the two subgroups were different, Subgroup 3 had 13.6% of accessions in 2010–2011, 10.4% in 2011–2012 and 6.4% overall that were significantly superior in yield to IS 18758 compared to 8.5% in 2010–2011, 3.8% in 2011–2012 and only 1.3% in overall in both seasons in Subgroup 2. For 100-seed weight also, a similar pattern was observed with Subgroup 3 showing a greater number of significant accessions (52, 62, and 54) than Subgroup 2 (15, 21, and 14) over the control IS 18758. The two post-rainy seasons had a differential influence on the accessions in both the subgroups. Combined over both seasons, the grain yield varied from 10 to 54 g in Subgroup 2 and 7 to 49 g in Subgroup 3, with a mean value of 24 g for both the Subgroups.

The performance of the top 15 accessions with a desirable combination of DFL, grain yield and 100-seed weight in the Subgroups 2 and 3 are given in Table 4. The selected 15 Subgroup 2 sources on average yielded 33 g, 11.4% more than the control IS 18758 (29 g). IS 4698 from India with 54 g was the best line in this group, giving 86.2% more grain yield than IS 18758. Other promising high-yielding sources in this group were IS 22294 from Botswana, IS 29241 and IS 29187 from Swaziland and IS 13971 from South Africa, producing 20.6–24.1% more grain yields than IS 18758 (Table 4). In Subgroup 3, the 15 selected accessions yielded on average 38 g, 31% more than IS 18758. Of these, IS 1004 from India, and IS 23586 and IS 23590 from Ethiopia (40–49 g), and IS 31706, IS 23891, and IS 28141 (39–42 g) from Yemen were significantly superior and gave 34–69.0% more yield than IS 18758. IS 31706, IS 23891, and IS 28141 had a good combination of significantly greater yields (39–42 g) and 100 seed weight (4.69–5.33 g) than the control IS 18758 (yield 29 g, 100-seed weight 2.37 g).

### Performance of PTS Mini Core Sources

The 19 PTS accessions originated from 9 countries, predominated by China with 8 accessions followed by 2 each from Korea, Zimbabwe and South Africa and 1 each from Cameroon, Yemen, Swaziland, the United States and Myanmar (Table 3). The grain yield of 19 PTS ranged from 2 to 29 g in the 2010–2011, 3–33 g in the 2011–2012 and 2–28 g overall in both the seasons. None of the PTS accessions was significantly better than control IS 18758, however, IS 30451 was promising in 2010–2011, 2011–2012 and combined over two post-rainy seasons with grain yields similar to IS 18758 (29 g) (Table 4).

### Distribution of Sensitive and Insensitive Sources Across Latitudes

**FIGURE 1:**
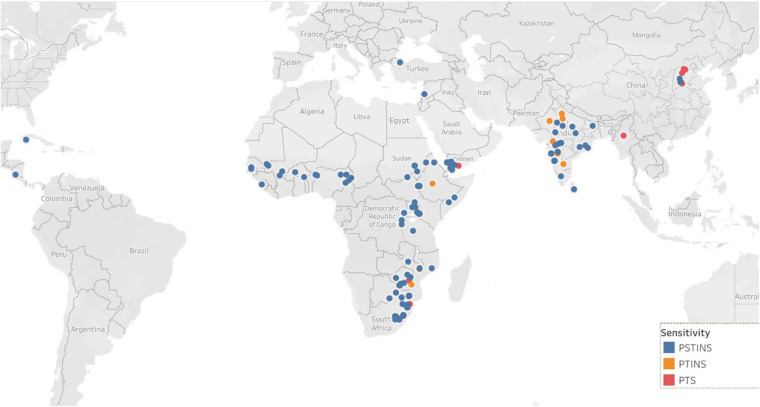
Distribution of sorghum mini core accessions with known geographical coordinates.

Data on the latitude of origin of only 135 mini core accessions, including 11 PTINS, 10 PTS, and 114 PSTINS landraces were available and plotted in Figure 1. For the 103 mini core landraces, the hemispheres were decided based on country of origin and the four landraces which originated from the countries spread over both the hemisphere were not included in this analysis. Of the 80 PSTINS of Subgroup 2, latitude data was available only for 41 and in Subgroup 3 on 73 out of 125. Hemisphere wise, 86 accessions (7 PTINS, 71 PSTINS, and 8 PTS) were from North Hemisphere and 49 (4 PTINS, 43 PSTINS, and 2 PTS) were from South Hemisphere. Among PSTINS distribution of Subgroup 2 and 3 was contrasting, with Subgroup 2 being predominant in the South Hemisphere (29 out of 41) and Subgroup 3 being predominant in the North (59 out of 73). The PTINS sources were distributed from 6 to 30° in the northern hemisphere and from 15° to 30° in the southern hemisphere. Similarly, the PTS sources were distributed from 10 to 40° in the north and from 15 to 30° in the southern hemisphere. Since the number of accessions in the northern and southern hemisphere in the PTINS and PTS was less than 10 in each, it would be meaningless to determine and discuss the patterns. In the case of PSTINS Subgroup 2, all the 29 sources in the southern hemisphere originated from 15 to 30° and all the 59 sources in Subgroup 3 originated from 0 to 30° in the northern hemisphere. The 12 sources of Subgroup 2 were distributed in northern from 11 to 45° and 14 sources in the southern hemisphere originated from 0 to 25°. In both the hemisphere, an increase in latitude results in a decrease in DFL and CGDD in the entire mini core collection. The mean yield among hemispheres was similar in the entire mini core, PTINS, PSTINS, Subgroup 3, and PTS. However, the range was higher in the entire mini core and PSTINS (7–54 g), Subgroup 2 (10–54), and 3 (7–49) in the northern hemisphere than in the southern hemisphere. A similar pattern was observed for the range of 100-seed weight (Table 5).

**TABLE 5:** Hemisphere wise, mean and range of days to 50% flowering, cumulative growing degree days (CGDD), plant height, grain yield and 100-seed weight of the entire mini core and sensitivity response groups.

Trait/group	Northern hemisphere				Southern hemisphere			
	Mean		Range		Mean		Range	
	Rainy	Post-rainy	Rainy	Post-rainy	Rainy	Post-rainy	Rainy	Post-rainy
**Entire mini core (160 accessions northern hemisphere; 77 accessions southern hemisphere)**								
Days to 50% flowering	87	71	44–148	51–120	73	76	49–143	58–100
CGDD	1430	863	756–2356	658–1485	1225	913	839–2289	728–1202
Plant height (cm)	315	238	129–446	120–369	270	229	115–399	106–313
Grain yield g plant^–1^		23		7–54		24		2–37
100-Seed weight (g)		2.53		1.00–5.33		2.2		0.48–3.42
**PTINS (11 accessions northern hemisphere; 7 accessions southern hemisphere)**								
Days to 50% flowering	69	69	57–84	56–87	69	69	61–82	58–78
CGDD	1159	840	971–1386	711–1041	1159	832	1025–1350	728–941
Plant height (cm)	283	224	185–335	129–331	270	215	189–335	137–260
Grain yield g plant^–1^		22		14–33		21		10–29
100-Seed weight (g)		2.20		1.48–2.89		2.14		1.43–2.99
**PSTINS (125 accessions northern hemisphere; 65 accessions southern hemisphere)**								
Days to 50% flowering	92	72	44–148	51–120	75	76	54–143	58–100
CGDD	1514	871	756–2356	658–1485	1246	913	910–2289	728–1202
Plant height (cm)	324	239	129–446	120–369	271	230	115–399	106–313
Grain yield g plant^–1^		24		7–54		25		9–37
100-Seed weight (g)		2.61		1.00–5.33		2.30		1.42–3.42
**PSTINS Subgroup 2 (36 accessions northern hemisphere; 44 accessions southern hemisphere)**								
Days to 50% flowering	58	69	44–83	54–93	61	74	54–78	62–92
CGDD	974	830	756–1369	685–1108	1037	888	910–1298	766–1096
Plant height (cm)	259	220	129–354	132–311	242	220	115–319	106–295
Grain yield g plant^–1^		22		10–54		27		15–36
100-Seed weight (g)		2.17		1.32–3.61		2.38		1.42–3.42
**PSTINS Subgroup 3 (99 accessions northern hemisphere; 21 accessions southern hemisphere)**								
Days to 50% flowering	104	73	61–148	51–120	103	80	66–143	58–100
CGDD	1710	887	1026–2356	658–1485	1683	964	1112–2289	728–1202
Plant height (cm)	347	246	192–446	120–369	331	250	211–399	166–313
Grain yield g plant^–1^		24		7–49		22		9–37
100-Seed weight (g)		2.77		1.00–5.33		2.13		1.42–3.21
**PTS (14 accessions northern hemisphere; 5 accessions southern hemisphere)**								
Days to 50% flowering	49	66	46–62	60–88	62	87	49–66	73–94
CGDD	832	804	785–1052	738–1054	1039	1037	839–1110	880–1121
Plant height (cm)	258	240	214–293	202–287	254	235	189–298	156–278
Grain yield g plant^–1^		20		14–28		18		2–25
100-Seed weight		1.99		1.33–2.43		1.70		0.48–2.50

### Correlations Among Quantitative Traits in the Insensitive and Sensitive Groups

Phenotypic correlations were performed among all quantitative traits in the entire mini core, in the PTINS, PSTINS and PTS groups and two subgroups of the PSTINS separately (Table 6). The DFL in the rainy and post-rainy seasons were highly correlated with plant height in the rainy and post-rainy seasons in the entire core, PTINS, entire PSTINS, PSTINS Subgroup 2 and Subgroup 3 (*p* < 0.001–0.05), but it was non-significant in the PTS group. Contrastingly, grain yield in the post-rainy season was highly correlated with DFL, CGDD and plant height in the rainy and post-rainy seasons (*r* = 0.302–0.413, *p* < 0.01–0.001) only in the PSTINS Subgroup 2 and non-significant in other groups except entire mini core and PSTINS entire group. In the latter two groups, the entire mini core and PSTINS group the magnitude of correlations was low at 0.187 and 0.190, respectively. Further, in the PTS group the highly significant correlations were observed only between 100-seed weight and grain yield in post-rainy, plant height between rainy and post-rainy, and DFL in the rainy and post-rainy seasons, all other correlations were non-significant (Table 6). This demonstrated that the strength of correlations depended upon the population used under study. The more homogenous the group, the better were the estimate of correlation. Further, the proportion of variance in one trait that can be attributed to its linear relationship with a second trait is indicated by the square of the correlation coefficient (Snedecor and Cochran, 1980). Estimates of this value greater than 0.71 or lower than −0.71 have been suggested as meaningful correlations (Skinner et al., 1999). In our study, we found such high correlation coefficients between plant height in the rainy and post-rainy seasons in all six groups (Table 6).

**TABLE 6:** Correlations among different traits in the entire mini core collection and sensitivity response groups.

Trait		DF-R^†^	DF-PR	CGDD-R	CGDD-PR	PLHT-R	PLHT-PR	GY-PR
DF-PR	Entire (242)	0.485***						
	PTINS (18)	0.927***						
	PSTINS (205)	0.492***						
	Sub-group 2 (80)	0.860***						
	Sub-group 3 (125)	0.591***						
	PTS (19)	0.976***						
CGDD-R	Entire (242)	1.000***	0.484***					
	PTINS (18)	0.999***	0.927***					
	PSTINS (205)	1.000***	0.491***					
	Sub-group 2 (80)	0.994***	0.861***					
	Sub-group 3 (125)	1.000***	0.593***					
	PTS (19)	1.000***	0.979***					
CGDD-PR	Entire (242)	0.489***	0.997***	0.488***				
	PTINS (18)	0.934***	0.996***	0.934***				
	PSTINS (205)	0.495***	0.997***	0.494***				
	Sub-group 2 (80)	0.850***	0.997***	0.849***				
	Sub-group 3 (125)	0.583***	0.997***	0.585***				
	PTS (19)	0.976***	1.000***	0.979***				
PLHT-R	Entire (242)	0.744***	0.326***	0.745***	0.327***			
	PTINS (18)	0.510*	0.541*	0.515*	0.495*			
	PSTINS (205)	0.745***	0.320***	0.747***	0.324***			
	Sub-group 2 (80)	0.427***	0.415***	0.410***	0.416***			
	Sub-group 3 (125)	0.520***	0.256**	0.526***	0.252**			
	PTS (19)	0.119	0.132	0.123	0.139			
PLHT-PR	Entire (242)	0.473***	0.570***	0.472***	0.559***	0.724***		
	PTINS (18)	0.705***	0.817***	0.710***	0.794***	0.838***		
	PSTINS (205)	0.517***	0.582***	0.517***	0.573***	0.735***		
	Sub-group 2 (80)	0.352***	0.487***	0.344**	0.489***	0.873***		
	Sub-group 3 (125)	0.582***	0.612***	0.586***	0.596***	0.731***		
	PTS (19)	0.09	0.155	0.098	0.165	0.896***		
GY-PR	Entire (242)	0.088	0.119	0.089	0.09	0.109	0.187**	
	PTINS (18)	0.34	0.466	0.336	0.434	0.145	0.242	
	PSTINS (205)	0.02	0.092	0.021	0.061	0.067	0.190**	
	Sub-group 2 (80)	0.379***	0.401***	0.394***	0.387***	0.302**	0.413***	
	Sub-group 3 (125)	0.027	−0.015	0.027	−0.047	0.029	0.104	
	PTS (19)	0.182	0.047	0.174	0.046	0.148	0.023	
HSW-PR	Entire (242)	0.239***	−0.203***	0.239***	−0.204***	0.229***	−0.012	0.492***
	PTINS (18)	−0.118	0.014	−0.118	−0.013	−0.229	−0.153	0.747***
	PSTINS (205)	0.180**	−0.248***	0.180**	−0.247***	0.207**	−0.003	0.454***
	Sub-group 2 (80)	−0.049	−0.077	−0.04	−0.065	−0.106	−0.171	0.237*
	Sub-group 3 (125)	0.02	−0.350***	0.016	−0.353***	0.151	−0.034	0.553***
	PTS (19)	0.009	−0.16	−0.006	−0.161	−0.058	−0.267	0.698***

^†^DF-R, days to 50% flowering in rainy; DF-PR, days to 50% flowering in post-rainy; CGDD-R, cumulative growing degree days in rainy; CGDD-PR, cumulative growing degree days in post-rainy; PLHT-R, Plant height (cm) in rainy; PLHT-PR, Plant height (cm) in post-rainy; GY-PR, Grain yield (g plant-1) in post-rainy; HSW-PR, 100-seed weight in post-rainy; *, **, *** significant at P ≤ 0.05, 0.01, 0.001, respectively.

## Discussion

The response of crops to the prevailing photoperiod and temperature conditions determines their adaptation to the wide range of different environments in which it is cultivated worldwide. Sorghum is a short-day species in which flowering initiation occurs only during the short-day condition (Oliveira et al., 2019). The majority of accessions conserved at the ICRISAT genebank do not flower or take much more time when they are being grown in the long-day rainy season (June–October) in India, while most accessions flower in the short-day post-rainy (October–February) season. In this study, the sorghum mini core accessions were characterized for traits related to photoperiod and temperature sensitivity, revealed a significant genotypic variation for DFL, CGDD, plant height, grain yield and 100-seed weight, and the interactions between genotype and season (year) were significant. This indicated that the sorghum mini core collection had adequate genotypic variation and the performance of genotypes differed in the two seasons.

Considering the DFL and CGDD, the mini core accessions were categorized into photoperiod and temperature insensitive (PTINS) (18 accessions), photoperiod sensitive and temperature insensitive (PSTINS) (205 accessions), and photoperiod and temperature-sensitive (PTS) (19 accessions) and identified best performing accessions in each of the group for their utilization in crop improvement. The 19 PTS sources identified in this study flowered 14–28 days earlier in the long-day rainy season than in the short-day post-rainy season, but with similar CGDD requirement in the two seasons. Among PSTINS accessions, 80 accessions were in Subgroup 2 that flowered 5–22 days earlier in the rainy season than in the post-rainy season, but with significantly higher CGDD requirement during the rainy season. These two groups (PTS and PSTINS subgroup 2) may have different mechanisms to trigger flowering than the 18 PTINS sources and the 125 PSTINS Subgroup 3 sources. The important question then is “Are there landraces in the global collections which are adapted to long-days such as PTS and PSTINS Subgroup 2 sources in the present study?” A critical analysis of flowering under controlled temperature and photoperiod conditions may provide the answer to whether flowering in some sorghum accessions is triggered by long-day or a combination of long-day × temperature interaction. In most photoperiod and temperature studies under controlled conditions, only a few cultivars have been used to model the flowering, which agrees with the known hypothesis that sorghum is a short-day plant, which may not be true when diverse landraces representing the diversity of global collection are used as shown in the present study with mini core collection.

In the entire collection of sorghum (Upadhyaya et al., 2018), and also in the mini core collection in this study, the mean and range of DGL and CGDD requirements for flowering decreased with the increase in latitude in both the hemisphere. The magnitude of the relationship between DFL and latitude differed between groups (PTINS, PSTINS, and PTS groups and two subgroups of the PSTINS separately) indicating that it varied with the constitution of the group. The differences in the relationship between northern and southern hemispheres in the mini core could be attributed to the differences in hemispheres in terms of the proportion of land and water and associated climatic parameters (Upadhyaya et al., 2018). The northern hemisphere has four times land portion (80%) than the ocean (20%), and land surfaces conduct the heat poorly, therefore, most of the absorbed radiation heats the land surface and associated vegetation in the northern hemisphere. In addition high levels of man-made pollution also contribute to the higher temperature in the northern hemisphere. The southern hemisphere has one-and-half times ocean portion (60%) than land (40%), and water is a good conductor of heat, and most of the radiation absorbed is stored in the deeper waters in the southern hemisphere.

Plant breeders in their quest to develop high-yielding cultivars with a broad adaptation need to work on several traits simultaneously. It is therefore important to have sources that facilitate developing such cultivars. In our efforts toward identifying such sources, this study was undertaken to identify photoperiod and temperature insensitive sources in the sorghum mini core collection. In earlier studies, sorghum mini core accessions have been evaluated for biotic (Sharma et al., 2010; Radwan et al., 2011; Seifers et al., 2012; Borphukan, 2014) and abiotic (Kapanigowda et al., 2013; Upadhyaya et al., 2016c, 2017) stresses, and for grain nutritional (Upadhyaya et al., 2016a) and bioenergy traits (Wang et al., 2011; Upadhyaya et al., 2014a). Several photoperiod- and/or temperature-insensitive sources identified in this study has also been reported as sources of other important traits for the breeding program. Of the 18 PTINS sources, 15 have been reported as sources for one or more traits. IS 5094 has been reported tolerant to drought based on the drought tolerance index (Upadhyaya et al., 2017), resistant to downy mildew (Sharma et al., 2010), charcoal rot, stem borer and shoofly. Similarly, IS 473, another PTINS was found resistant to grain mold, Anthracnose, leaf blight and rust (Sharma et al., 2010, 2012). These and other PTINS sources with resistance could be good sources to breed sorghum cultivars with stress resistance and insensitivity to photoperiod and temperature to impart wide adaptation. A large number of PSTINS sources, 65 out of 80 in the Subgroup 2 and 89 out of 125 in Subgroup 3 had shown desirability to one or more traits. Four Subgroup 2 accessions, IS 602 (Zinc, germination at low temperature, grain mold, and charcoal rot), IS 1233 (Iron, Zinc, germination at low temperature, and grain mold), IS 4515 (drought, stem borer, shoot fly, and aphids), and IS 4698 (dual purpose, stem borer, shoot fly, and aphids) had desirability for four traits each. Similarly, three accessions, IS 22294 and IS 30092 for downy mildew, grain mold and charcoal rot and IS 12945 for grain mold, leaf blight and charcoal rot. These sources could be useful in developing resistant sorghum cultivars suitable for rainy season. The 89 Subgroup 3 sources had shown desirability for large number of traits. One line, IS 23684 for six traits (protein, Brix, anthracnose, leaf blight, rust, and Aphids), five accessions for four traits: IS 7305 for Saccharification yield, seedling vigor at low temperature, germination at low temperature, downy mildew; IS 23521 for Anthracnose, leaf blight, rust and Aphids; IS 23992 for iron, downy mildew, grain mold, and stem borer; IS 24939 for Brix, Anthracnose, leaf blight, and Aphids and IS 31557 for downy mildew, leaf blight, charcoal rot, and Aphids. These and other sources in this group would be a valuable resource for breeders to develop multipurpose sorghum cultivars with adaptation to the post-rainy season. Among the 19 PTS sources, 15 have been identified as desirable for 1–5 traits. Two accessions for five traits, IS 1212 for Iron, Zinc, seedling vigor at low temperature, downy mildew and grain mold and IS 24503 for Iron, Zinc, leaf blight, rust, and charcoal rot. IS 29314 (germination at low temperature, downy mildew, and aphids), IS 30466 (Zinc, downy mildew, and charcoal rot) and IS 30507 (Iron, Zinc, and charcoal rot) were good for three traits. These and other sources in this group could be useful sources to the breeders to develop cultivars suitable for the areas where both photoperiod and temperatures are important in sorghum cultivation and can be exploited to enhance production.

The use of novel variability is the key to continued progress in crop improvement and for widening the genetic base of the newly bred cultivars (Upadhyaya et al., 2009). The information on the differential response of sorghum accessions in response to photoperiod and temperature sensitivity could be useful in the sorghum improvement program. The PTINS, PTS and PSTINS sources identified in this study could help breeders to use appropriate sources in their program. The photoperiod and temperature insensitive accessions can be utilized to developed cultivars with broader adaptation, while the highly photoperiod sensitive tall accessions can be utilized for biomass and forage improvement. Further, the information on the desirability of the identified accessions for other important traits will be useful to determine the accessions based on needs for extensive multilocation evaluation in the targeted environments to identify stable sources for breeding programs. The source accessions identified in the present study could be utilized in genetic and genomic investigations to understand the mechanism underlying photoperiod and temperature response and mapping the genes. Sorghum researchers can obtain seed samples of these accessions from the ICRISAT genebank for research purposes through a Standard Material Transfer Agreement (SMTA).

## Data Availability Statement

The original contributions generated for this study are included in the article/Supplementary Material, further inquiries can be directed to the corresponding author.

## Author Contributions

HU and MV: conceptualization. MV: data analysis and curation. HU: writing – original draft. HU, MV, and VA: writing – review and editing. All authors contributed to the article and approved the submitted version.

## Conflict of Interest

The authors declare that the research was conducted in the absence of any commercial or financial relationships that could be construed as a potential conflict of interest.

## Footnotes

1 http://intranet.icrisat.org/IcrisatLocal/weather.aspx

2 https://www.vsni.co.uk/
