# Supplementary material for: Variation for Photoperiod and Temperature Sensitivity in the Global Mini Core Collection of Sorghum
Source: Front Plant Sci. 2021 Jun 29;12:571243. doi: 10.3389/fpls.2021.571243 (PMC8276039; doi:10.3389/fpls.2021.571243)
Supplement: Supplementary file 1 [file Table_1.DOCX]

**Table S1.** Estimation of genotypic, season, genotype x season variance components based on combined of two rainy seasons and two postrainy seasons.

| Season and trait |  | Variance component | | Wald's statistics for Year | Mean | SE(d) | LSD (P≤0.05) | CV (%) |
| --- | --- | --- | --- | --- | --- | --- | --- | --- |
|  |  | Genotype | Genotype × Year |  |  |  |  |  |
| Rainy season (combined of 2010 & 2011) | | | | | | | | |
| Days to 50% flowering |  | 756.46** | 15.13** | 277.28** | 83 | 2.45 | 4.8 | 4.18 |
| Cumulative growing degree days (CGDD) |  | 185081.00** | 4074.0** | 152.38** | 1371 | 39.88 | 78.17 | 4.11 |
| Plant height (cm) |  | 3938.6** | 682.10** | 191.08** | 301 | 16 | 31.36 | 7.51 |
|  |  |  |  |  |  |  |  |  |
| Postrainy (combined of 2010-1 & 2011-12) | | | | | | | | |
| Group 1 (33 accessions) |  |  |  |  |  |  |  |  |
| Days to 50% flowering |  | 41.54** | 11.99** | 7.24* | 62 | 1.44 | 2.83 | 2.31 |
| Cumulative growing degree days (CGDD) |  | 4184.6** | 1223.7** | 227.59** | 767 | 15.09 | 29.57 | 1.97 |
| Plant height (cm) |  | 1670.71** | 189.57** | 167.53** | 197 | 9.3 | 18.24 | 4.73 |
| Grain yield per plant (g) |  | 73.47** | 28.00** | 45.77** | 21 | 3.73 | 7.32 | 18.02 |
| 100-seed weight |  | 0.33** | 0.06** | 0.01ns | 2.67 | 0.23 | 0.46 | 8.72 |
|  |  |  |  |  |  |  |  |  |
| Group 2 (82 accessions) |  |  |  |  |  |  |  |  |
| Days to 50% flowering |  | 18.09** | 10.79** | 25.15** | 67 | 1.49 | 2.91 | 2.21 |
| Cumulative growing degree days (CGDD) |  | 2051.7** | 135.1** | 589.69** | 815 | 15.64 | 30.65 | 1.92 |
| Plant height (cm) |  | 1369.7** | 144.0** | 323.18** | 220 | 11.44 | 22.42 | 5.21 |
| Grain yield per plant (g) |  | 47.68** | 18.90** | 46.57** | 23 | 4 | 7.85 | 17.39 |
| 100-seed weight |  | 0.54** | 0.07** | 13.68** | 2.47 | 0.22 | 0.44 | 9.07 |
|  |  |  |  |  |  |  |  |  |
| Group 3 (88 accessions) |  |  |  |  |  |  |  |  |
| Days to 50% flowering |  | 18.09** | 10.79** | 25.15** | 75 | 1.49 | 2.91 | 2.21 |
| Cumulative growing degree days (CGDD) |  | 2051.7** | 135.1** | 589.69** | 903 | 15.64 | 30.65 | 1.92 |
| Plant height (cm) |  | 1369.7** | 144.0** | 323.18** | 247 | 11.44 | 22.42 | 5.21 |
| Grain yield per plant (g) |  | 47.68** | 18.90** | 46.57** | 25 | 4 | 7.85 | 17.39 |
| 100-seed weight |  | 0.54** | 0.07** | 13.68** | 2.43 | 0.22 | 0.44 | 9.07 |
|  |  |  |  |  |  |  |  |  |
| Group 4 (31 accessions) |  |  |  |  |  |  |  |  |
| Days to 50% flowering |  | 38.56** | 13.01** | 62.30** | 86 | 1.57 | 3.07 | 1.82 |
| Cumulative growing degree days (CGDD) |  | 5323.8** | 1997.2** | 366.69** | 1030 | 18.12 | 35.51 | 1.76 |
| Plant height (cm) |  | 2252.8** | 103.8** | 138.2** | 275 | 11.32 | 22.19 | 4.12 |
| Grain yield per plant (g) |  | 81.38** | 15.71** | 22.55** | 23 | 4.74 | 9.28 | 20.75 |
| 100-seed weight |  | 0.41** | 0.03** | 6.47* | 2.19 | 0.14 | 0.28 | 6.46 |
|  |  |  |  |  |  |  |  |  |
| Group 5 (8 accessions) |  |  |  |  |  |  |  |  |
| Days to 50% flowering |  | 161.49* | 33.40* | 14.44** | 97 | 1.39 | 2.72 | 1.43 |
| Cumulative growing degree days (CGDD) |  | 26105.0* | 5565.8* | 55.89** | 1165 | 16.91 | 33.15 | 1.45 |
| Plant height (cm) |  | 1663.5* | 153.6ns | 81.77** | 271 | 13.93 | 27.31 | 5.13 |
| Grain yield per plant (g) |  | 88.75* | 4.24ns | 20.46** | 21 | 7.18 | 14.08 | 33.46 |
| 100-seed weight |  | 0.42* | 0.09ns | 0.19ns | 1.97 | 0.16 | 0.31 | 8.03 |

*,** Significant at P≤0.05 and P≤0.01, respectively
